# Feasibility and Safety of Evaluating Patients with Prior Coronary Artery Disease Using an Accelerated Diagnostic Algorithm in a Chest Pain Unit

**DOI:** 10.1371/journal.pone.0163501

**Published:** 2016-09-26

**Authors:** Roy Beigel, Alexander Fardman, Ronen Goldkorn, Orly Goitein, Sagit Ben-Zekery, Nir Shlomo, Michael Narodetsky, Moran Livne, Avi Sabbag, Elad Asher, Shlomi Matetzky, PLatelets And Thrombosis In Sheba (PLATIS) study group

**Affiliations:** The Leviev Heart Center, Sheba Medical Center, Tel-Hashomer, affiliated to The Sackler School of Medicine, Tel-Aviv University, Tel Aviv, Israel; Kurume University School of Medicine, JAPAN

## Abstract

An accelerated diagnostic protocol for evaluating low-risk patients with acute chest pain in a cardiologist-based chest pain unit (CPU) is widely employed today. However, limited data exist regarding the feasibility of such an algorithm for patients with a history of prior coronary artery disease (CAD). The aim of the current study was to assess the feasibility and safety of evaluating patients with a history of prior CAD using an accelerated diagnostic protocol. We evaluated 1,220 consecutive patients presenting with acute chest pain and hospitalized in our CPU. Patients were stratified according to whether they had a history of prior CAD or not. The primary composite outcome was defined as a composite of readmission due to chest pain, acute coronary syndrome, coronary revascularization, or death during a 60-day follow-up period. Overall, 268 (22%) patients had a history of prior CAD. Non-invasive evaluation was performed in 1,112 (91%) patients. While patients with a history of prior CAD had more comorbidities, the two study groups were similar regarding hospitalization rates (9% vs. 13%, p = 0.08), coronary angiography (13% vs. 11%, p = 0.41), and revascularization (6.5% vs. 5.7%, p = 0.8) performed during CPU evaluation. At 60-days the primary endpoint was observed in 12 (1.6%) and 6 (3.2%) patients without and with a history of prior CAD, respectively (p = 0.836). No mortalities were recorded. To conclude, Patients with a history of prior CAD can be expeditiously and safely evaluated using an accelerated diagnostic protocol in a CPU with outcomes not differing from patients without such a history.

## Introduction

Chest pain is the second most common cause for emergency department visits in adults and is responsible for nearly 6 million visits annually in the United States alone [1]. While over 2 million of these chest pain patients are further admitted to hospital, coronary etiology is found in only about one-third of them [1–3]. In order to limit hospitalization costs on the one hand and to avoid misdiagnosis of discharged patients with acute chest pain on the other, the concept of using an accelerated diagnostic protocol in a chest pain unit (CPU) has evolved over the last two decades. These CPU diagnostic protocols have proven to be safe and cost-effective in evaluating low-risk patients with acute chest pain and are widely employed today [4–9]. Since patients with a history of prior coronary artery disease (CAD) presenting with acute chest pain are usually categorized as intermediate or even high risk for an acute coronary syndrome [9], they are frequently excluded from being rapidly evaluated in a CPU. Currently, there are limited data regarding the safety of applying an accelerated diagnostic protocol and evaluating patients with a history of prior CAD in a CPU. The aim of our study was to assess the feasibility and safety of evaluating patients with a history of prior CAD in a CPU, and compare our results with those of patients without a history of prior CAD.

## Material and Methods

Our study included a prospective cohort of 1,220 consecutive patients who presented to our emergency department with complaints of chest pain, suggestive of coronary origin, and underwent further evaluation in our CPU. Inclusion and exclusion criteria for CPU admission have been previously described [7]. Briefly, these included all patients with unremarkable electrocardiograms (ECGs) (either normal or unchanged from a previous ECG), and a negative troponin test on admission to the CPU. Patients admitted to the CPU were evaluated via an accelerated diagnostic protocol which consists of an observation period of 12 hours using a computerized ST-segment monitoring system, followed by a repeat ECG and cardiac troponin measurements. Patients who demonstrated any of the following symptoms during the observation period: ischemic ECG changes, repeated elevated troponin levels, ST changes, or ongoing chest pain of possible ischemic origin, were admitted for further evaluation. Patients without any of the above-mentioned findings during the observation period were further evaluated by the attending cardiologist which decided regarding the need for non-invasive evaluation by either myocardial perfusion scintigraphy (MPS), multi-detector computed tomography (MDCT), or stress echocardiography. All tests results were reviewed by the staff cardiologists or roentgenologists. Patients with a negative evaluation were discharged, while those with more than mild ischemia on MPS, a narrowing of an epicardial coronary artery ≥50% upon MDCT, or the appearance of a new regional wall motion abnormality by stress echocardiography, were further referred for invasive coronary angiography.

All patients admitted to the CPU were considered eligible for either MPS or stress echocardiography and underwent evaluation according to test availability. However, exclusion criteria for undergoing MDCT were as follows: (1) age over 70 years; (2) prior CAD; (3) weight more than120 kg; (4) absence of sinus rhythm; (5) known contraindication to iodine contrast; (6) abnormal renal function (serum creatinine more than1.4 mg/dl). In selected cases, according to the discretion of the attending cardiologist, patients with a history of prior CAD were eligible for evaluation by the MDCT if not meeting additional exclusion criteria. Prior CAD was defined as either a history of prior myocardial infarction, prior percutaneous coronary intervention, coronary artery bypass grafting, or the presence of known atherosclerotic CAD by coronary angiography or MDCT prior to the current hospitalization.

The composite outcome of our study was defined as follows: hospitalization due to chest pain, acute coronary syndrome, coronary revascularization (either by percutaneous intervention or bypass graft), and/or death during 60 days post discharge from the CPU. Additionally, the safety of evaluation of patients with a history of prior CAD in the CPU was assessed by the rates of hospitalization, angiography, revascularization and death during evaluation in the CPU. Follow-up was obtained either by a visit to the outpatient clinic or by a pre-specified telephone interview performed at least 60 days post discharge. We reviewed records from the Ministry of the Interior in order to ascertain vital status regarding patients lost to follow-up. The study has been conducted according to the principles expressed in the declaration of Helsinki. As this was a retrospective analysis, whenever follow-up data was not available through patients' medical records, oral informed consent was obtained from the participants to obtain follow-up data, which was recorded on a prespecified form. Patients were notified about the study by mail and telephone and were given the option to opt out prior to participation. The Sheba medical center IRB approved the study and the informed consent process. All data were analyzed using SPSS software 20 (SPSS, Inc., Chicago, Illinois, USA). Categorical variables were compared using chi-square tests. Two-tailed Student’s independent *t*-tests were used for comparison of continuous variables. A statistically significant difference was considered as a p value less than 0.05. Parameters that were found to be significantly different, between those patients with a history of prior CAD and those without, were inserted into the outcome analysis, which was performed using a multivariable logistic regression model.

## Results

The study included 1,220 consecutive patients who presented to the emergency department with acute chest pain suggestive of coronary origin and were evaluated in the CPU. Overall, 268 (22%) patients had a history of prior CAD. Baseline characteristics of the study population are shown in Table 1. Patients with prior CAD, compared with those without known CAD, were older, more likely to be male, and to have hypertension, diabetes mellitus, dyslipidemia, peripheral vascular disease, and a prior stroke.

**Table 1:** Baseline characteristics of the study cohort.

Variable	All patients (N = 1,220)	Patient without prior CAD (N = 952)	Patient with prior CAD (N = 268)	P^a^ value
Age (years, mean ± SD)	55±12	54±12	60±12	<0.001
Males (n, %)	849 (70)	616 (65)	233 (87)	<0.001
Hypertension (n, %)	519 (43)	347 (37)	172 (64)	<0.001
Diabetes mellitus (n, %)	207 (17)	142 (15)	65 (24)	<0.001
Dyslipidemia (n, %)	666 (55)	463 (49)	203 (76)	<0.001
Family history (n, %)	315 (26)	269 (28)	46 (17)	<0.001
Prior CVA/TIA (n, %)	36 (3)	22 (2.3)	14 (5.2)	0.01
Prior PVD (n, %)	13 (1.1)	7 (0.7)	6 (2.2)	0.05

CVA, Cerebrovascular accident; TIA, Transient ischemic attack; PVD, Peripheral vascular disease

^a^P value for comparison between patients with and without a prior history of coronary artery disease.

### Observation period/CPU course

During the observation period in the CPU, 58 patients (4.7%) were hospitalized, of whom 45 (78%) were diagnosed with suspected cardiac symptoms (Table 2). Patients with a history of prior CAD, compared with those without prior CAD, had more episodes of ongoing chest pain presumed to be of cardiac origin (3.4% vs. 0.9%, p< 0.01), more episodes of elevated troponin levels in subsequent tests (4.1% vs. 0.6%, p value <0.001), and consequently were more likely to be diagnosed as having an acute coronary syndrome requiring hospitalization (9% vs. 2.2%, p value < 0.01).

**Table 2:** Outcomes of patients during the observation period in the chest pain unit.

Variable	All patients (N = 1,220)	Patients without prior CAD (N = 952)	Patients with prior CAD (N = 268)	P^a^ value
Hospitalized without non-invasive test for suspected cardiac cause (n, %)	45 (3.7)	21 (2.2)	24 (9)	<0.001
Ongoing chest pain (n, %)	18 (1.5)	9 (0.9)	9 (3.4)	0.008
ST/ECG changes (n, %)	18 (1.5)	11 (1.2)	7 (2.6)	0.09
Positive troponin (n, %)	17 (1.4)	6 (0.6)	11 (4.1)	<0.001
Angiography (n, %)	45 (3.7)	21 (2.2)	24 (9)	<0.001
Re-vascularization (n, %)	25 (2.1)	10 (1.1)	15 (5.6)	<0.001
Hospitalized without non-invasive test for non-cardiac cause (n, %)	13 (1)	6 (0.6)	7 (2.6)	0.01

CAD, Coronary artery disease; ECG, Electrocardiogram

^**a**^ P value for comparison between patients with and without a history of prior coronary artery disease.

### Non-invasive evaluation

**Fig 1:**
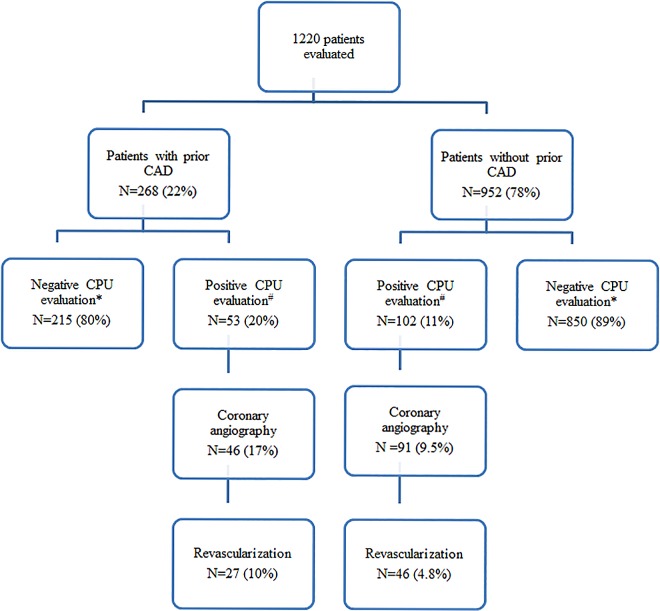
Patient evaluation flow chart. * Patients with a negative evaluation were discharged. ^#^ Positive evaluation includes: hospitalization during the observation period in the chest pain unit and patients with a positive non-invasive tests who were hospitalized for further investigation. CAD, Coronary artery disease; CPU, Chest pain unit

A patient evaluation flow chart is shown in Fig 1. Fifty patients (4.1%) who had an uneventful course during the observation period in the CPU were discharged after clinical evaluation without further non-invasive testing. Fourteen patients (28%) had a history of prior CAD and 36 (72%) did not. The remaining 1,112 (91%) patients underwent non-invasive testing: 623 patients (56%) MPS, 423 (38%) MDCT, and 66 (6%) stress echocardiography. Of these 1,112 patients, 1002 (90%) were discharged without further investigation. None of the patients died during the evaluation period in the CPU.

**Fig 2:**
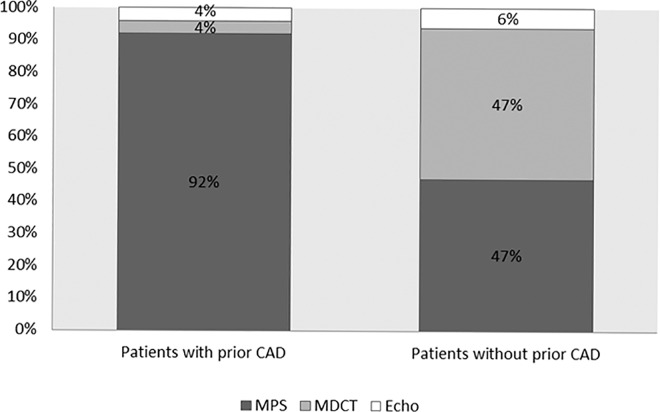
Distribution of imaging tests between the study groups. MPS, Myocardial perfusion scintigraphy; MDCT, Multidetector computed tomography; Echo, Stress echocardiography; CAD, Coronary artery disease.

As shown in Fig 2, patients without a history of prior CAD underwent significantly more evaluation tests with MDCT than with MPS, compared with those patients who had a history of prior CAD (47% vs. 4%, p value < 0.001 for MDCT, 47% vs. 92%, p value <0.001 for MPS). When comparing between patients with and without a history of prior CAD, both groups were similar regarding discharge after non-invasive evaluation (91% vs. 87%, p = 0.08, respectively). Furthermore, there was no difference between the two groups regarding hospitalization rates (9% vs. 13%, p = 0.08), coronary angiography (13% vs. 11%, p = 0.4), and revascularization (6% vs. 5.2%, p = 0.7) (Table 3). Overall, 73 patients underwent revascularization during hospitalization in the CPU. Of them, 25 patients underwent revascularization during the observation period prior to non-invasive evaluation, and 48 patients after undergoing non-invasive evaluation.

**Table 3:** Outcomes of patients who underwent non-invasive evaluation in the chest pain unit.

Variable	All Patients (N = 1,112)	Patients without prior CAD (N = 889)	Patients with prior CAD (N = 223)	P^a^ value
Patients discharged from chest pain unit (n, %)	1002 (90)	808 (91)	194 (87)	0.08
Patients hospitalized (n, %)	110 (10)	81 (9)	29 (13)	0.08
Coronary angiography (n, %)	92 (12)	70 (13)	22 (11)	0.41
Percutaneous coronary intervention (n, %)	44 (6)	33 (6)	11 (5.2)	0.7
Coronary artery bypass grafting (n, %)	4 (0.5)	3 (0.5)	1 (0.5)	1
Death (n, %)	0	0	0	-

CAD, Coronary artery disease

^a^ P value for comparison between patients with and without a history of prior coronary artery disease

### Sixty-day follow-up

A complete 60-day follow-up was available for 912 (91%) of the 1,002 patients discharged from the CPU. Following a review of records from the Ministry of the Interior, we were able to verify that all the remaining 90 patients (9%) were alive during the 60-day follow-up period. Eighteen patients (2%) met the composite outcome (Table 4). There was no difference between the two study groups in the incidence of the 60-day primary composite outcome (1.6% vs. 3.2%, respectively, p = 0.8 for patients without versus those with a history of prior CAD). This result remained unaffected also after adjustment for confounding risk factors including sex, age, hypertension, diabetes mellitus, dyslipidemia, family history of CAD, peripheral vascular disease, and a history of prior stroke using multivariable logistic regression modeling (OR 1.12, 95% CI 0.37–3.3). Similarly, there were no significant differences in each of the individual components of the composite outcome including readmissions (1.5% vs. 2.7%, p = 0.35) and acute coronary syndrome (0.14% vs. 1.1%, p = 0.12). There was a trend towards more revascularization (0.9% vs. 2.1%, p = 0.07) in the prior CAD group (Table 4). None of the patients died during the follow-up period.

**Table 4:** Outcome of patients at 60-day follow-up.

Event type	All Patients (N = 912)	Patients without prior CAD (N = 727)	Patients with prior CAD (N = 185)	Odds Ratio	Confidence interval (95%)	P^a^ value
Primary composite outcome (n, %)	18 (2)	12 (1.6)	6 (3.2)	1.12	0.37–3.3	0.83^*^
Re-admission due to chest pain (n, %)	16 (1.7)	11 (1.5)	5 (2.7)			0.35
Acute coronary syndrome (n, %)	3 (0.32)	1 (0.14)	2 (1.08)			0.12
PCI or CABG (n, %)	8 (0.88)	4 (0.55)	4 (2.1)			0.07
Death (n, %)	0	0	0			

^a^Fisher’s exact test p value.

*P value is based on multivariable logistic regression incorporating age, sex, hypertension, diabetes mellitus, hyperlipidemia, family history of coronary artery disease, prior peripheral vascular disease, and prior cerebral vascular accidents.

CAD, Coronary artery disease; PCI, Percutaneous coronary intervention; CABG, Coronary artery bypass graft

## Discussion

In the current study, in a relatively large, prospective group of more than 1,200 patients with acute chest pain, we have shown that patients with prior CAD can be safely and expeditiously evaluated using an accelerated diagnostic protocol in a CPU. To the best of our knowledge, this is the largest cohort to report such outcomes in patients with a history of prior CAD undergoing a rapid CPU evaluation. There are, however, a limited number of studies that have evaluated smaller cohorts reaching conclusions similar to ours [10–12].

The majority of patients in the current study had an uneventful course during the observation period in the CPU, with less than 5% of them hospitalized due to supporting evidence of an evolving acute coronary syndrome. The finding of significantly more hospitalizations in the group of patients with a prior history of CAD compared with those patients without a history of prior CAD is not surprising considering the higher risk profile and proven presence of CAD in the former group of patients [13]. Nevertheless, almost 90% of patients with a history of prior CAD had an uneventful course during the observation period and were able to complete an accelerated non-invasive evaluation while in the CPU. Furthermore, patients hospitalized in the CPU received prompt medical attention, as well as continuous monitoring, and were immediately referred to coronary angiography and revascularization when indicated, thereby avoiding adverse outcomes. Of those patients hospitalized, a similar percent in both groups underwent revascularization (6.5% vs. 5.7%, p = 0.748 for patients without versus those with a history of prior CAD, respectively).

The vast majority (91%) of patients in our cohort underwent non-invasive evaluation, which is the cornerstone of the decision making algorithm for patients in the CPU. By appropriate utilization of non-invasive modalities and despite the significant difference in baseline characteristics of the two patient groups, comparable outcomes of non-invasive evaluation were recorded. The majority of patients were discharged from the CPU (91% vs. 87%, p = 0.082 for patients without versus those with a history of prior CAD, respectively), demonstrating similar outcomes in both patient groups. As we [8] and others [4–7] have previously shown, the utilization of an accelerated diagnostic algorithm in a CPU facilitates a reduction in hospitalization costs and length of stay on the one hand, while diminishing misdiagnosis of discharged patients with acute chest pain on the other. During the 60-day follow-up period, both groups had a similar low rate of the prespecified composite outcome as well as individual secondary endpoints, thus confirming the reliability of non-invasive evaluation of patients with a history of prior CAD in a CPU.

Because our study presents “real-life” data, it includes a non-randomized cohort of patients which could introduce an unwanted bias. However, the two study groups were similar regarding primary composite outcome, results which were constant after controlling for confounding risk factors using logistic regression modeling. The ability of our study to detect the differences between the study groups may be underpowered by the low incidence of the primary outcome. As such, this study is only a hypothesis, and further randomized prospective trials are warranted in order to validate our results.

## Conclusion

Patients with a history of prior CAD can be safely and expeditiously evaluated using an accelerated diagnostic protocol in a CPU with outcomes that do not differ from those of patients without a history of prior CAD.
